# Construction and application of a heterogeneous quality control library for the Xpert MTB/RIF assay in tuberculosis diagnosis

**DOI:** 10.3389/fcimb.2023.1128337

**Published:** 2023-03-17

**Authors:** Zehao Guan, Xuefei Han, Weigang Huang, Xueliang Wang, Hualiang Wang, Yun Fan

**Affiliations:** ^1^ R&D Laboratory of Quality Control Material, Shanghai Center for Clinical Laboratory, Shanghai, China; ^2^ Shanghai Academy of Experimental Medicine, Shanghai, China

**Keywords:** *Mycobacterium tuberculosis*, Xpert MTB/RIF assay, quality control material, proficiency testing, heterogeneous host

## Abstract

Proficiency testing based on quality control materials is an important component of the quality assurance system for detection methods. However, in the detection of infectious diseases, it is a challenge to use quality control materials derived from clinical samples or pathogens owing to their infectious nature. The Xpert MTB/RIF assay, endorsed by the World Health Organization, is one of the most widely implemented assays in the detection of *Mycobacterium tuberculosis* along with rifampicin resistance and its heterogeneity. Clinical isolates are typically used as quality controls for this assay, leading to concerns about biosafety, constrained target sequence polymorphisms, and time-consuming preparation. In this study, a heterogeneous quality control library for the Xpert MTB/RIF assay was constructed based on DNA synthesis and site-directed mutation, which provides sufficient rifampicin resistance polymorphisms, enabling monitoring all five probes of Xpert MTB/RIF and its combinations. *Escherichia coli* and *Bacillus subtilis* were used as heterogeneous hosts rather than the pathogen itself to eliminate biosafety risks; thus, preparation does not require a biosafety level III laboratory and the production time is reduced from a few months to a few days. The panel was stable for more than 15 months stored at 4°C and could be distributed at room temperature. All 11 laboratories in Shanghai participating in a pilot survey identified the specimens with corresponding probe patterns, and discordant results highlighted inappropriate operations in the process. Collectively, we show, for the first time, that this library, based on heterogeneous hosts, is an appropriate alternative for *M. tuberculosis* detection.

## Introduction

Tuberculosis (TB), caused by *Mycobacterium tuberculosis*, is the leading lethal infection, responsible for more than 1 million deaths globally each year in the last decade. Low- and middle-income countries have the highest impact, accounting for 98% of all TB cases (World Health Organization, 2021). Drug resistance and diagnosis delay are two key challenges in effectively treating the pathogen (Dartois and Rubin, 2022; Dong et al., 2022; Liebenberg et al., 2022). In 2019, cases of development of rifampicin resistance or multiple drug resistance represented nearly half a million cases (World Health Organization, 2020a; World Health Organization, 2021). A timely diagnosis is essential for early initiation of appropriate therapy, thereby preventing drug resistance transmission and improving the treatment outcomes. However, conventional culture or phenotypic drug-susceptibility testing is time-consuming, requiring 10 weeks or longer (World Health Organization, 2011). The delay to rifampicin-resistant detection was reported to be 62 days in Shanghai, China (Wu et al., 2020). The Xpert MTB/RIF assay (Cepheid, Sunnyvale, CA, USA) is the game changer towards addressing this issue, targeting the 81-bp rifampicin resistance-determining region (RRDR) of the *rpoB* gene and identifying *M. tuberculosis* along with rifampicin resistance within only 2 h, while simultaneously reflecting RRDR heterogeneity associated with variable levels of rifampicin resistance (Shea et al., 2021; Li et al., 2022) *via* various patterns of its five probes (A-E) and recombination (World Health Organization, 2011; Uddin et al., 2020). By integrating sample processing in the cartridge, less operational skill is required, which especially favors resource-limited regions. With these superiorities, the Xpert MTB/RIF assay has been one of the most widely implemented assays to date, being used in more than 122 TB high-burden developing countries (Albert et al., 2016) and having been recommended as an initial test in adults and children with signs and symptoms of TB by the World Health Organization (2020b).

Like all diagnostic tests, a quality assurance program based on a proficiency test panel is required to ensure the quality of the Xpert MTB/RIF assay; nevertheless, the development of proficiency test panels has not kept pace with the expansion of Xpert MTB/RIF testing. A limited number of panels are available, most of them derived from *M. tuberculosis*, including clinical isolates with drug resistance (Scott et al., 2011; Scott et al., 2014; Gumma et al., 2019), which raises important biosafety concerns and constraints in transportation, especially cross-border transportation. The infectious nature of the pathogen imposes specific infrastructure requirements. A biosafety level III laboratory and associated equipment are prerequisites for preparing these panels (Gumma et al., 2019; Klein et al., 2020), which are not easily accessed in resource-limited settings, hindering the local manufacture of the proficiency test panels, especially in resource-limited regions and countries that are severely affected by TB. In practice, however, local manufactures are encouraged to improve the performance quality of the assay by reducing costs and output time, while ensuring sustainability (Gumma et al., 2019). Another critical issue needing attention is that *M. tuberculosis* has an extremely slow growth rate, leading to a period of several months in production and inactivation verification (Scott et al., 2011; Gumma et al., 2019). The long processing period prevents easy access to these panels. Furthermore, the current available panels lack variety in both rifampicin resistance polymorphisms (corresponding probe patterns in Xpert MTB/RIF) and bacterial load settings for a designated panel. Typically, the panel contains several specimens with similar bacterial loads, including a rifampicin-susceptive specimen and one or two rifampicin-resistant specimens isolated from clinical strains (Scott et al., 2014; Gumma et al., 2019) with the resistance polymorphisms confined to the dominating strains of a local epidemic. Only very few of the probe patterns in Xpert MTB/RIF can be expected in these isolates. The custom panel based on dominant strains from one region may not be suitable for all outbreak areas and could fail to reflect subdominant strains in the region.

These limitations can be attributed to the pathogen’s infectious nature and extremely slow growth rate, as well as to the low numbers of strains with rifampin resistance polymorphisms collected for proficiency tests. A synthetic biology methodology—involving non-pathogenic heterogeneous hosts harboring target sequences simulating *M. tuberculosis*—seems to be a promising solution. However, few studies have investigated this issue. Scott et al. (2014), compared five external quality assessment (EQA) panels for Xpert MTB/RIF with a scoring system across various qualitative and quantitative variables. The panel based on *M. tuberculosis* DNA encapsulated in the heterogeneous host *Escherichia coli* yielded the lowest score owing to the requirement of a cold chain for transport and the inconvenience in dispensing the samples into the cartridge. Mitigating these pitfalls may make the method efficient and suitable for use in low-income countries. An improved panel based on a heterogeneous host library was developed in our laboratory. The library harbored mutants corresponding to various probe patterns of Xpert MTB/RIF for the resistance heterogeneity detection. Two non-pathogenic bacilli, *E. coli* and *Bacillus subtilis*, served as heterogeneous hosts. In this study, we investigated the features of this library and its application feasibility in the Xpert MTB/RIF assay quality control.

## Materials and methods

### Preparation of the mutant library based on *E. coli*

A DNA fragment, MTB-RIF-S (**Table S1**), containing the 81-bp core region of the *rpoB* gene and a partial 16S rDNA sequence of *M. tuberculosis* separated by EcoRI and HindIII recognition sites, was synthesized and ligated into the pUC57 plasmid, which was digested by EcoRV-BamHI to yield the plasmid p16S-S and strain EC-16S-S. The DNA fragment MTB-RIF-BDE was synthesized in the same manner as described above, including three-nucleotide mutants corresponding to probes B, D, and E of the Xpert MTB/RIF assay, yielding plasmid p16S-BDE and strain EC-16S-BDE. These two plasmids were constructed by Sangon Biotech (Shanghai, China). To remove the 16S rDNA region, p16S-S and p16S-BDE were digested by HindIII and self-ligated, yielding pRIF-S and pRIF-BDE, respectively. The plasmid series was constructed by PCR-based mutation from these two plasmids.

PCR-induced mutagenesis was performed using QuikChange Site-Directed Mutagenesis Kit (Agilent, CA USA) according to the manufacturer instructions, with some modifications. Briefly, PrimeSTAR HS DNA polymerase (Takara Japan) was used instead of Pfu Turbo DNA polymerase, and the PCR mixture of 50 μl included 100–300 ng templates, 0.3–1 μM primer pair, 200 μM dNTPs, and 1.5 U of DNA polymerase. The extension reaction was initiated by pre-heating the reaction mixture to 98°C for 10 s, followed by 30 cycles of 98°C for 10 s and 68°C for 4 min, and incubation at 68°C for 10 min. The PCR-amplified products were purified and treated with the restriction enzyme DpnI (Takara Japan). One microliter of the product was transformed into *E. coli* DH5α competent cells and inoculated on Luria-Bertani (LB) agar plates with 100 mg/ml ampicillin. The mutants were identified by sequencing. The primers and templates used for each plasmid are listed in **Tables S2** and **S3**, respectively.

### Preparation of the mutant library based on *E. coli* and *B. subtilis*

The fragment containing the 81-bp *rpoB* core region and partial 16S rDNA sequence was amplified from p16S-S with primer 1 (flanked with a BtsI-v2 recognition site) and primer 2 (**Table S2**), further digested with BtsI-v2, and ligated into the shuttle plasmid pBE980a (digested with BtsI-v2 and NheI and then treated with the Klenow large fragment) to yield pBE-MTB-S.

The mutation plasmids were constructed as described above for the *E. coli* host with the primers and templates listed in **Tables 1** and **S2**, respectively, except that 50 μg/ml kanamycin was used to screen the transformants. The shuttle plasmids were transferred to *B. subtilis* WB600 (a gift from Professor Zhiqun Lu) to prepare the mutation library based on *B. subtilis via* electroporation as described by Xue et al. (1999) with minor modifications. Trehalose (0.5 M) was added to the electroporation medium (0.5 M sorbitol, 0.5 M mannitol, 0.5 M trehalose, and 10% glycerol); the competent cells in a 1-mm electroporation cuvette were shocked using a pulser (Gene Pulser Xcell Total System; Bio-Rad, Hercules, CA, USA) at 2100 V, 25 μF, and 200 Ω; and 50 μg/ml kanamycin was used for selection of the transformants.

**Table 1:** Mutant strain based on *B. subtilis*.

Strains	Plasmids	Templates	Primers (see Table S2)
BS-S	pBE-MTB-S	\	\
BS-A	pBE-MTB-A	pBE-MTB-S	MA-F&MA-R
BS-B	pBE-MTB-B	pBE-MTB-S	MB-F&MB-R
BS-C	pBE-MTB-C	pBE-MTB-S	MC-F&MC-R
BS-D	pBE-MTB-D	pBE-MTB-S	MD-F&MD-R
BS-E	pBE-MTB-E	pBE-MTB-S	ME-F&ME-R
BS-AD	pBE-MTB-AD	pBE-MTB-D	MA-F&MA-R
BS-AE	pBE-MTB-AE	pBE-MTB-A	ME-F&ME-R
BS-BD	pBE-MTB-BD	pBE-MTB-S	MBD-F&MB-R
BS-BE	pBE-MTB-BE	pBE-MTB-E	MB-F&MB-R
BS-DE	pBE-MTB-DE	pBE-MTB-E	MD-F&MD-R
BS-BDE	pBE-MTB-BDE	pBE-MTB-E	MBD-F&MB-R
BS-ADE	pBE-MTB-ADE	pBE-MTB-DE	MA-F&MA-R

### Sample preparation and panel distribution for the survey

A pilot survey based on the *E. coli* library was performed involving 11 participating laboratories of Shanghai, including two major TB-designated medical institutions (Shanghai Pulmonary Hospital and Shanghai Public Health Clinical Center) that are responsible for most of the diagnosis and treatment of TB in Shanghai, using the 10 GeneXpert Dx and 1 Infinity System devices.

To prepare the panel sample, the fresh colony was inoculated into 3 ml LB medium with corresponding antibiotics and incubated at 37°C overnight. The next day, the culture was centrifuged, washed, and diluted with sterile water to reach a final absorbance value (optical density at 600 nm [OD_600_]) of 1 as the stock strain. The strain was then diluted to preset concentrations with 1 mM Tris-HCl, 1% sodium carboxymethyl cellulose, 10% glycerol, and 0.2% KroVin 600 (Seebio, Shanghai, China) as an antibacterial agent, and distributed at 1 ml per tube for storage at 4°C. The survey panel consisted of three samples varying in OD value: (1) RIF-S (OD_600_ = 10^-4^), (2) RIF-BDE (OD_600_ = 10^-7^), and (3) RIF-E (OD_600_ = 10^-5^), corresponding to the wild type, the most predominant mutation, and a rare triple mutation found in India (Thirumurugan et al., 2015), respectively. These panels were couriered or hand-delivered to the participating Xpert laboratories in Shanghai. The detection reports in PDF file format generated by GeneXpert software were collected for analysis.

### Stability at room temperature

RIF-S samples with OD_600_ = 10^-5^ were prepared as described above; stored at room temperature (in a range of 20–30°C) for 0, 7, 10, and 20 days, respectively; and then stored at 4°C until tested in the Xpert system in triplicate. A similar test was performed at 37°C for 10 days.

### Storage stability of the panel

The survey panel samples were stored at 4°C for 15 months, subjected to the Xpert MTB/RIF assay in triplicate, and compared with the results of the survey.

### Chemical inactivation of bacteria with KroVin 600

KroVin 600 was used as a preservative to prolong the shelf lifetime of the panel. Meanwhile, to prevent unintended proliferation and the spread of host strains, a sterility test (bactericidal activity of KroVin 600) was performed as follows. Panel samples with *E. coli* DH5α or *B. subtilis* WB600 (OD_600_ = 1) were stored at 4°C, and 100 μl aliquots were spread on LB plates at irregular intervals following preparation for incubation at 37°C to detect the growth of bacteria.

To exclude the impaction of KroVin 600 on the test results, samples (RIF-S or BS-S, OD600 = 10-5) with or without Krovin 600 were tested with the Xpert system in triplicate. No significant differences were found (**Figure S1**).

### Ct difference between the two chassis cells

Strains RIF-S and BS-S prepared at OD_600_ of 10^-3^, 10^-5^, and 10^-7^ were stored at 4°C for more than three days and then tested with the Xpert system in triplicate.

### Statistical analysis

The means and standard deviations were calculated for the Ct quantitative variable for probe A. Microsoft Excel was used for all calculations, and an unpaired *t*-test was used for statistical comparisons in analyzing the stability of the specimens; *p* < 0.05 was considered statistically significant.

## Results

### RRDR library based on *E. coli*

**Figure 1:**
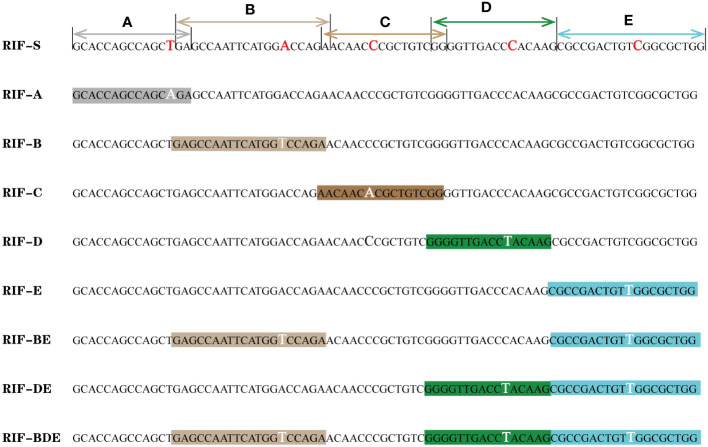
*rpo*B mutant library based on *E. coli*. **(A–E)**: probes of the Xpert MTB/RIF assay; the mutant and corresponding nucleotides are highlighted.

**Figure 2:**
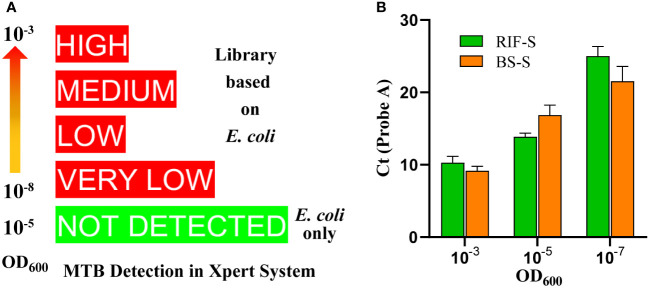
Quantitative and semi-quantitative results according to optical density values at 600 nm (OD_600_). **(A)** Semi-quantitative readings from the library based on (*E*) *coli* in the Xpert MTB/RIF assay, showing that OD_600_ of specimens ranges from 10^-8^ to 10^-3^. The rank is associated with the mutants and lots. **(B)** Cycle threshold (Ct) values of probe A for (*E*) *coli* and (*B*) *subtilis* with the same OD_600_ value.

An RRDR library was constructed based on *E. coli* (**Figure 1**), derived from two synthetic plasmids subjected to site-directed mutation with the aid of five primer pairs (**Table S3**). The library harbors mutants corresponding to the nine probe patterns of the Xpert MTB/RIF assay, including the wild type of the core region (RIF-S), five mutants targeting each probe of Xpert MTB/RIF (RIF-A, B, C, D, and E), two mutants corresponding to double probes (RIF-BE, RIF-DE), and one mutant corresponding to triple probes (RIF-BDE). These patterns were confirmed by the GeneXpert system with off-target effects in single, double, or triple probes reported as rifampicin-susceptible or -resistant *M. tuberculosis* with genotypic heterogeneity, whereas the *E. coli* chassis cell alone did not exhibit corresponding patterns (**Figures S2**, **S3**). In addition, all semi-quantitative scores reported for *M. tuberculosis* and detected by Xpert MTB/RIF (very low, low, medium, or high) were achieved by adjusting the bacterial OD value (**Figures 2A**, **S2**, **S3**).

### Stability of RRDR library samples at room temperature

**Figure 3:**
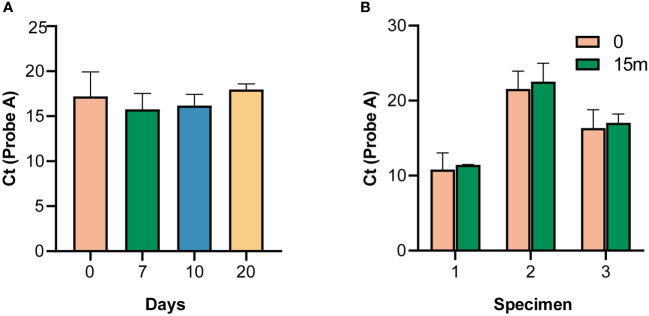
Stability of the panels. **(A)** Stability of specimens at room temperature. **(B)** Stability of specimens after 15 months of storage, according to the cycle threshold (Ct) value of probe A.

The specimens were stable for either more than 20 days at room temperature (**Figure 3A**), or more than 10 days at 37°C (**Figure S4**), indicating that a cold chain would not be required for transport and delivery. Typically, it takes no more than three days to deliver samples to most cities in China by courier.

### EQA *via* detection of discordance among laboratories

All participating laboratories (except for site 7) detected *M. tuberculosis* in all samples and identified rifampicin resistance in specimens 2 and 3, with the heterogeneity between them responding to different probe patterns; probes B, D, and E showed off-target effects in specimen 2, whereas probe E was missing in specimen 3 (**Table 2**).

**Table 2:** Discordant results identified in 11 laboratory sites participating in the survey.

	Specimen 1 (RIF-S)				Specimen 2 (RIF-BDE)				Specimen 3 (RIF-E)			
Site	MTB	Rif	Probes	Ct_A_	MTB	Rif	Probes	Ct_A_	MTB	Rif	Probes	Ct_A_
**1**	High	–	All	10.4	Medium	+	AC	19.3	High	+	ABCD	13.6
**2**	High	–	All	10	Low	+	AC	24.9	Medium	+	ABCD	16.6
**3**	High	–	All	10.5	Medium	+	AC	21.4	High	+	ABCD	13.6
**4**	High	–	All	9.6	Low	+	AC	22.2	High	+	ABCD	14.7
**5**	High	–	All	11.3	Medium	+	AC	21.6	High	+	ABCD	15.6
**6**	High	–	All	12.7	Low	+	AC	26	Medium	+	ABCD	16.9
**7**	Medium*	–	All	16.7	NEG*	\	\	\	Medium	+	ABCD	18.8
**8**	High	–	All	9.8	Medium	+	AC	21.7	High	+	ABCD	14.7
**9**	High	–	All	9.6	Medium	+	AC	18.8	Medium	+	ABCD	16.7
**10**	High	–	All	8.7	Medium	+	AC	19.2	Low*	+	ABCD	22.1
**11**	High	–	All	9.6	Medium	+	AC	20.4	Medium	+	ABCD	16.4
			Mean	10.8			Mean	21.6			Mean	16.3

*Discordant results.

MTB, Mycobacterium tuberculosis; Rif, rifampicin resistance; Probes, positive probes; Ct_A_, cycle threshold of Probe A; +, rifampicin resistance detected; – rifampicin resistance not detected; \, data not available; NEG, M. tuberculosis not detected.

**Figure 4:**
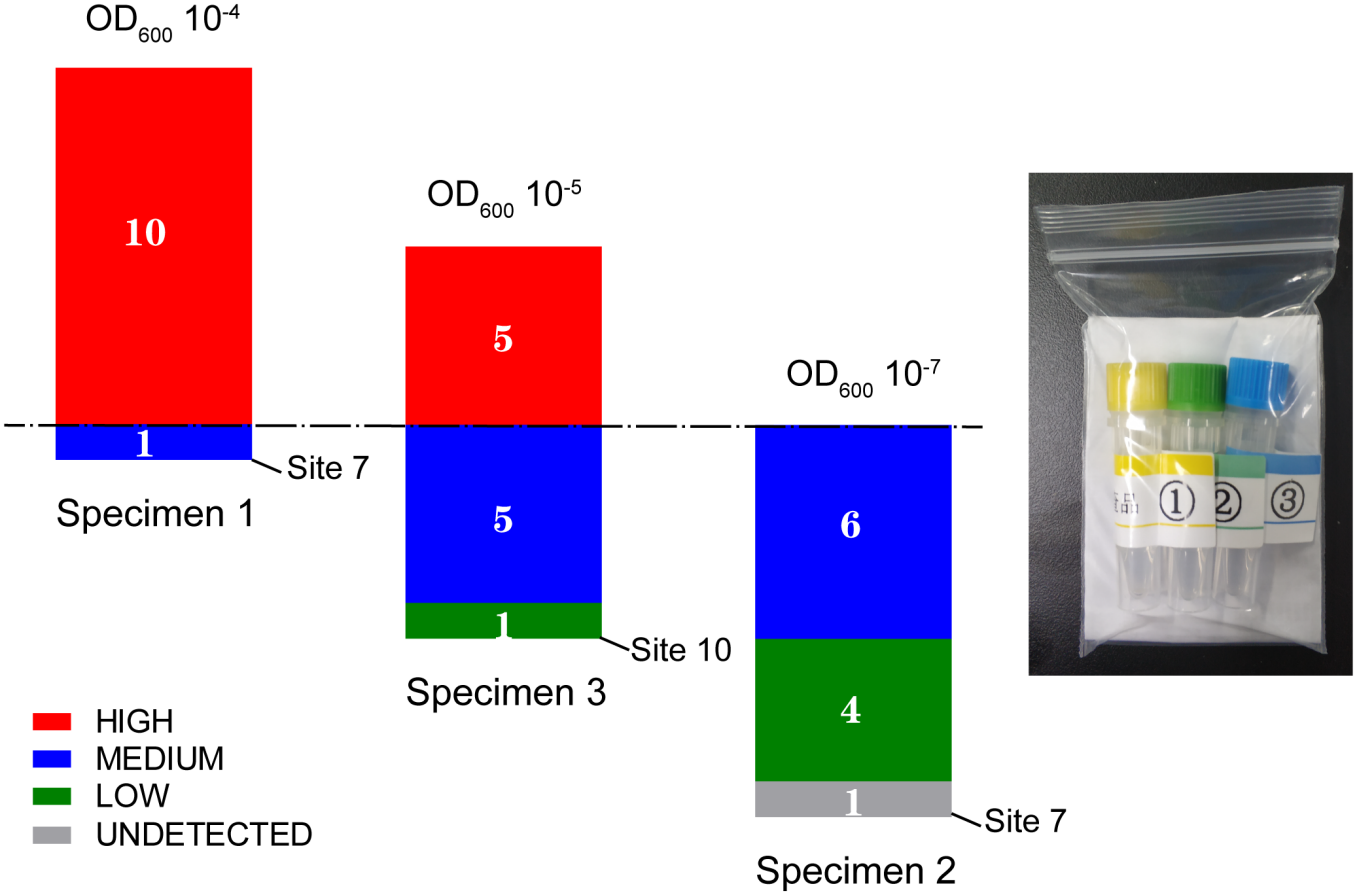
*M. tuberculosis* detection results of three specimens in the panel from the pilot survey in 11 clinical laboratories in Shanghai.

With respect to the semi-quantitative results in TB detection, most of the sites obtained the same or an adjacent rank for a given specimen (**Figure 4**). According to the dominant results, specimens 1, 2, and 3 were designated as “high,” “medium/low,” and “high/medium,” respectively. The hierarchy was confirmed by the mean Ct values of the probes of 10.8, 21.6, and 16.3 corresponding to specimens 1, 2, and 3, respectively, using the Ct of probe A as reference; a value of 22 is set as the threshold value between “low” and “medium” and a value of 16 is set as the threshold between “medium” and “high.” This ranking also showed a positive association with the OD values of the bacillus (**Table 2** and **Figure 4**).

Sites 7 and 10 were identified to have discordant results among the 11 participating laboratories. Site 7 failed to detect TB in specimen 2 and reported a semi-quantitative ranking of “medium” rather than “high” for specimen 1. Site 10 reported a result of “low” rather than “high/medium” for specimen 3 (**Figure 4**). Telephone interviews were conducted for these sites to understand the nature of this discordance. Site 10 reported sample loss for specimen 3 in the interview. Site 7 indicated that they had followed the procedure for sputum sediment rather than for raw sputum as performed at the other sites, which involves additional steps, including NaOH treatment, that are not used in the raw sputum procedure. To verify that these procedural differences were the causes of the discordance, three more samples of specimen 2 were retested at Site 7 following the raw sputum procedure, resulting in a report of a “medium” rank (mean Ct_pA_ = 21.4).

### Long-term stability

The survey panel was stable for more than 15 months at 4 °C, as indicated by the Ct values of probe A compared with the survey results (*p* > 0.05) (**Figure 3B**).

### Library expansion and chassis switch

A library based on the shuttle plasmid pBE980a was first prepared in *E. coli* DH5α. The pattern number was then expanded to 13 by introducing an additional primer and extra combinations of primers and templates based on site-directed mutation. The *B. subtilis* library was then constructed after transformation, containing 12 mutants and one wild-type sequence in the RRDR (**Table 1**), which was confirmed by the GeneXpert system with a corresponding probe pattern. Similar to the results for the *E. coli* chassis, no *M. tuberculosis* was detected with *B. subtilis* only (**Figure S5**). When comparing the two chassis harboring the same genotype of RRDR (BS-S vs. RIF-S), similar Ct values were obtained in the set OD values (**Figure 2B**). Neither *E. coli* nor *B. subtilis* colonies developed on the plates after 3 days of treatment with the panel matrix. These results indicated similar features of the library based on both bacteria, demonstrating their suitability in the preparation and application as quality controls for the Xpert MTB/RIF assay.

## Discussion

In this study, we developed a heterogeneous quality control library for the Xpert MTB/RIF assay with advantages of convenient preparation methods and accessibility. The library harbors mutants with sufficient RRDR polymorphisms in the Xpert MTB/RIF assay, enabling monitoring of its five probes and their combinations. The mutations in RRDR, responsible for the rifampicin resistance mechanism in 95% of cases (Helb et al., 2010; Uddin et al., 2020), are reported as single, double, or triple off-target of five probes or their combinations (Zaw et al., 2018). The mutant frequencies corresponding to different probes vary across regions, being very rare in some areas. Collecting strains with an appropriate diversity of probe patterns in the Xpert MTB/RIF assay for proficiency testing requires obtaining numerous rifampicin-resistant isolates. Out of 90 rifampicin-resistant isolates in India, only five distinct patterns in the assay are expected: three single mutations, one double mutation, and one triple mutation (Mokrousov et al., 2003). Similarly, out of 100 rifampicin-resistant isolates from 518 *M. tuberculosis* clinical strains identified in Shaanxi province of China, seven patterns of the Xpert system are expected (Yang et al., 2021). The pattern number expanded to nine with 205 rifampicin-resistant isolates identified in Bangladesh, including five single mutations, four double mutations, and no triple mutation (Uddin et al., 2020). Although the diverse mutation pattern suggests geographic variation (Zaw et al., 2018), customs and delivery constraints for transporting pathogens prevent access to obtain foreign isolates, thus confining the diversity possible to only locally available clinical strains. To overcome these barriers in ensuring appropriate EQA, the panel developed in this study includes 12 types of mutations corresponding to 12 distinct probe patterns in the Xpert MTB/RIF assay: five single mutations, five double mutations, and two triple mutations, with the aid of 11 primers. To our knowledge, this number exceeds the reported patterns expected in any survey performed to date, covering the probe patterns that can be expected in most TB-affected regions. More patterns that can be achieved by simply introducing additional primers, if needed, as shown in the study. Theoretically, the library can mimic any mutant in the core region, providing sufficient diversity of quality controls to customize panels for the designated EQA.

*B. subtilis* and *E. coli* were used as heterologous hosts for the library constructed in this study, and are extensively implemented as chassis for the biosynthesis of antibiotics (Liu et al., 2016), anti-tumor compounds (Tang et al., 2022), biomass (Liu et al., 2022), and therapeutics (Lynch et al., 2022), demonstrating good records regarding biosafety. Thus, a conventional laboratory is adequate for preparation of our panel, in contrast to the mandatory requirement of biosafety level III infrastructure sets for handling *M. tuberculosis*. This facilitates local manufacture of the panel in resource-limited countries, which also carry a high burden of TB. In addition, the slow-growth property of *M. tuberculosis* results in a time-consuming production process and subsequent inactivation verification. For confirmation of the inactivation of the pathogen, 42 days was required for dried culture spots using microbial growth incubation tubes (Becton, Dickinson, Sparks, MD, USA) (Scott et al., 2011) and 84 days was required for a dried tube specimen panel (Gumma et al., 2019). However, less than seven days was needed for our panels based on *B. subtilis* or *E. coli*, from inoculation to preparation to inactivation.

As shown in the pilot survey, the panel based on *E. coli* fully meets the EQA requirement for Xpert MTB/RIF by monitoring its probe patterns and responding to the concentration variation of the bacillus. The Ct values of the probes or semi-quantitative results of the panel were positively associated with bacterial OD_600_ values, offering a useful means in detecting discordance. Moreover, the OD value of bacilli can be easily adjusted and measured with a spectrophotometer, which is readily available in a conventional laboratory, even in resource-limited settings, compared with the requirement of more complex flow cytometry for existing *M. tuberculosis* panels (Scott et al., 2011). The requirement of a cold chain for transport and the inconvenience in dispensing the samples into the cartridge, two problems that plagued heterogeneous panels (Scott et al., 2014), has been resolved. Our panel involves a package fit for single use (**Figure 4**) and a matrix with appropriate fluidity; no transfer problems were reported in the present survey. Moreover, a cold chain is not required for allocation since the panel can be expected to remain stable, either for more than 20 days at room temperature, or 10 days at 37°C, which provides a particular advantage, especially for improving access to resource-limited regions and countries (Scott et al., 2014). The panel was stable for more than one year at 4°C and a tube of culture (3 ml) is adequate for conducting more than 10,000 tests, further demonstrating improved convenience and accessibility.

Although we demonstrated that the library based on *E. coli* or *B. subtilis* is suitable as a quality control material for the Xpert MTB/RIF assay, though the difference between the bacillus and mycobacteria chassis remains a potential concern, especially given their distinct cell wall features, which may affect DNA extraction (Picard et al., 2009). Mycobacteria are classified as gram-positive bacteria but with an outer membrane covering the cell wall, resembling the characteristics of gram-negative bacteria (Rohde, 2019). In this study, both gram-negative and gram-positive model bacteria, *E. coli* and *B. subtilis*, respectively, were tested as chassis cells; neither of these hosts prevented Xpert MTB/RIF from reading the mutant library. We speculate that the extraction procedure in the kit is sufficient for either *E. coli* or *B. subtilis* as well as for *M. tuberculosis*. It is worth noting that *Bacillus subtilis* subsp. *globigii*, a subspecies of *B. subtilis*, is used as the sample processing control in the Xpert MTB/RIF assay cartridge (Helb et al., 2010; World Health Organization, 2011). Nevertheless, a chassis that is more similar to *M. tuberculosis* would still be preferred. In light of the convenience in switching the chassis of the library, as shown in this study, it may be possible to use mutants based on *Mycobacterium smegmatis*, which is generally considered a non-pathogenic mycobacterium and grows faster than *M. tuberculosis* (Tyagi and Sharma, 2002; Sundarsingh et al., 2020); thus, we plan to test *M. smegmatis* as a possible chassis for this panel in further studies. Another discrepancy between the heterologous library and the target pathogen involves the vector of the target sequence. Multiple plasmid copies were used in construction of our library, whereas the pathogen harbors only one copy of the target sequence of Xpert MTB/RIF in the chromosome. Thus, further investigation is warranted to determine how this difference affects detection, and if a single-copy plasmid or integrative vector would be superior (such as providing better reproducibility in the “very low” rank). Importantly, successors of the Xpert MTB/RIF assay continue to expand the targeting sequences for various purposes such as detecting resistance to more drugs in the Xpert MTB/XDR assay (Naidoo and Dookie, 2022) and increasing the sensitivity of *M. tuberculosis* detection in the Xpert MTB/RIF Ultra assay, which incorporates the multicopy amplification targets “IS6110” and “IS1081” (Chakravorty et al., 2017). Thus, in developing the library for these assays, additional target sequences will need to be added as well as assigned to the vectors with a suitable copy number (single, multiple, or combined). Another limitation is that this pilot survey only covered a limited number of laboratories in Shanghai, China. As such, this study should be scaled up and performed in more rounds to more comprehensively investigate the features of the new library panel.

In summary, we have constructed a heterogeneous library for Xpert MTB/RIF assay quality control based on non-pathogenic bacteria, which overcomes the obstacles associated with the detected pathogen, including the biosafety risk, time-consuming preparation and verification, constrained laboratory infrastructure, and limited target sequence polymorphisms. The panel is based on a library that is suitable for applications in EQA and offers accessible quality control materials for the Xpert MTB/RIF assay, even in resource-limited regions, which tend to have higher TB burdens. Importantly, this work demonstrates the feasibility of the approach to use heterologous hosts as an alternative to pathogens, which can help to mitigate safety concerns and expand quality control for assays targeting infectious pathogens more broadly.

## Data availability statement

The original contributions presented in the study are included in the article/**Supplementary Material**. Further inquiries can be directed to the corresponding author.

## Author contributions

YF designed the study and performed the experiments and interpretation of data. XH and ZG conducted experiments of some representative bacteria. XW, HW, and WH contributed to part of the data analysis. All authors contributed to the article and approved the submitted version.

## Supplementary material

Supplementary Figure 1 Effect of KroVin 600 on the test results according to the cycle threshold (Ct) value of probe A.

Supplementary Figure 2 Xpert MTB/RIF assay results of the library based on *E. coli* (GeneXpert Dx System, partial). Mismatched probes are framed in rectangles.

Supplementary Figure 3 Xpert MTB/RIF assay results of the library based on *E. coli* (GeneXpert Dx System, Chinese version, partial). Mismatched probes are framed in rectangles.

Supplementary Figure 4 Stability of specimens at 37°C.

Supplementary Figure 5 Xpert MTB/RIF assay results of the library based on *B. subtilis* (GeneXpert Infinity System, partial). Mismatched probes are framed in rectangles.
