# Supplementary figures and images for: Construction and application of a heterogeneous quality control library for the Xpert MTB/RIF assay in tuberculosis diagnosis
Source: Front Cell Infect Microbiol. 2023 Mar 17;13:1128337. doi: 10.3389/fcimb.2023.1128337 (PMC10063913; doi:10.3389/fcimb.2023.1128337)

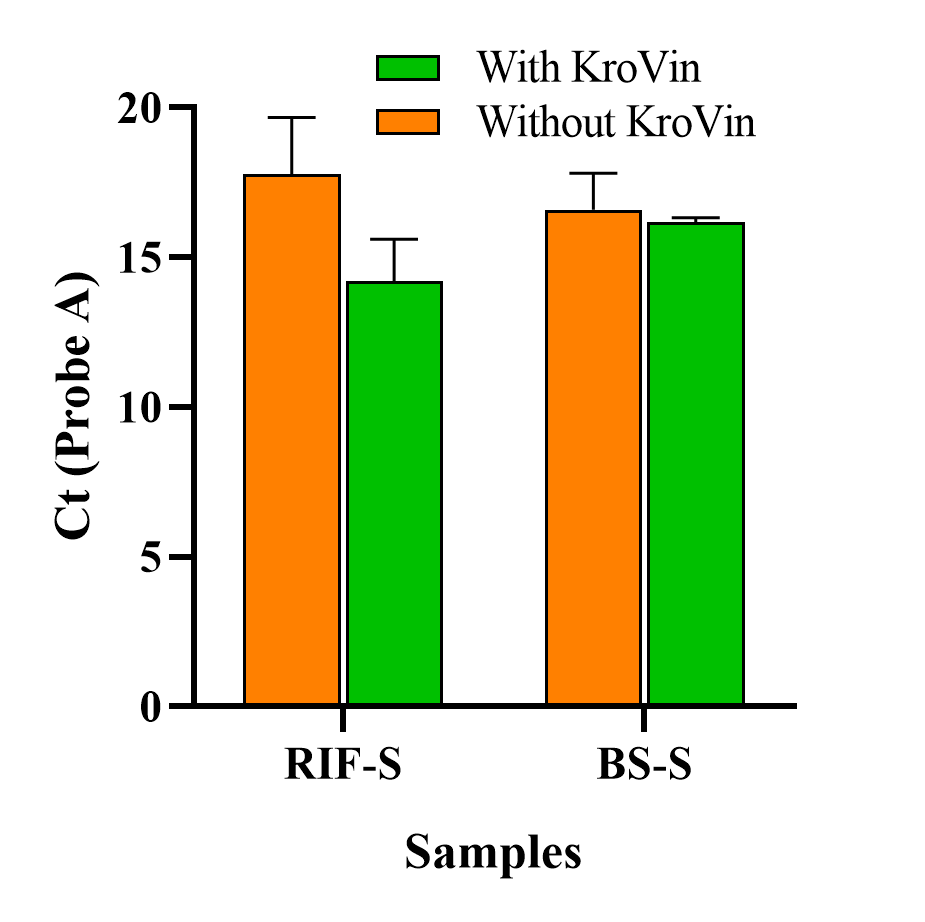

Supplement: Supplementary Figure 1 — Effect of KroVin 600 on the test results according to the cycle threshold (Ct) value of probe A. [file Image_1.tif]

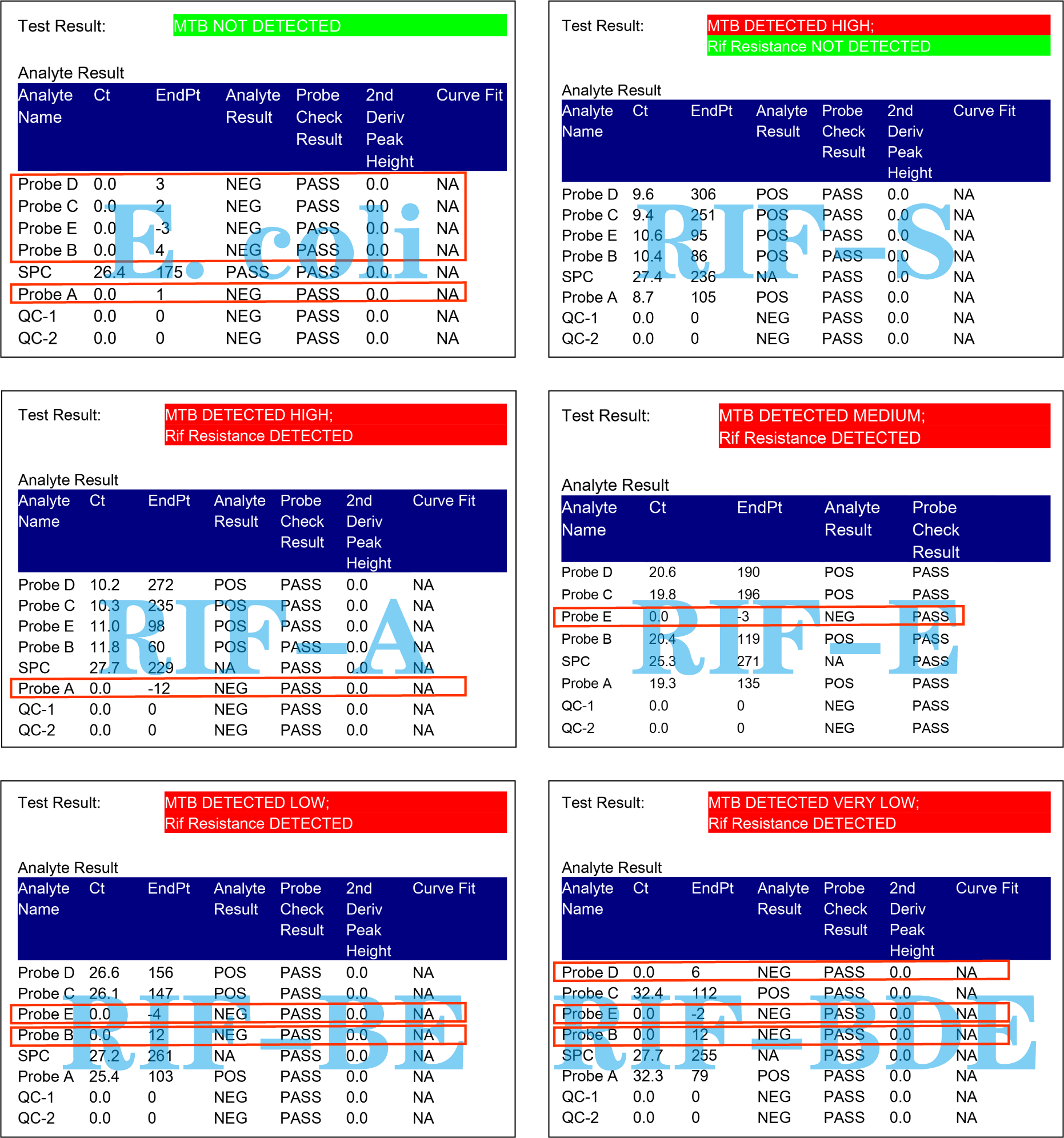

Supplement: Supplementary Figure 2 — Xpert MTB/RIF assay results of the library based on E. coli (GeneXpert Dx System, partial). Mismatched probes are framed in rectangles. [file Image_2.tif]

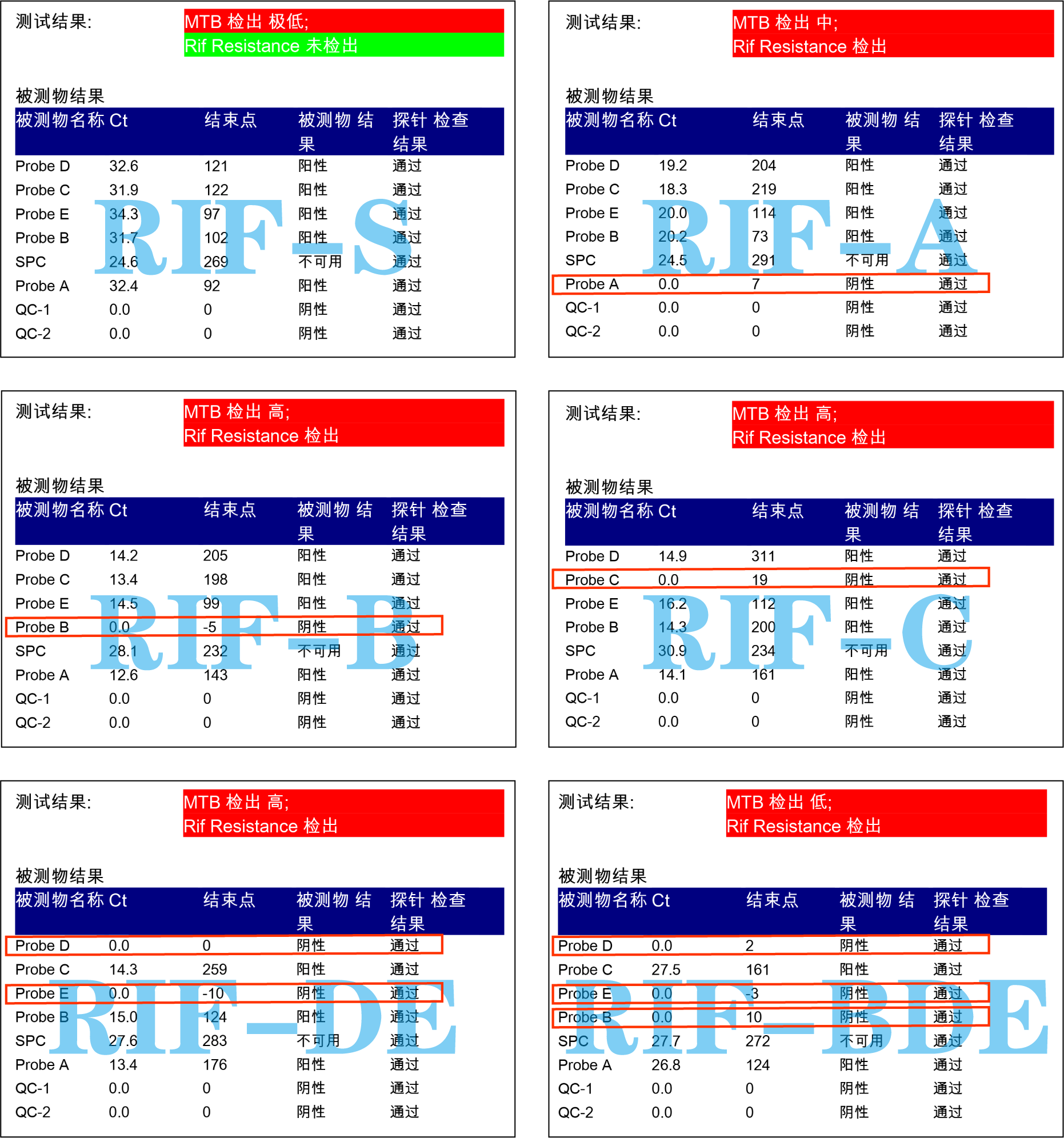

Supplement: Supplementary Figure 3 — Xpert MTB/RIF assay results of the library based on E. coli (GeneXpert Dx System, Chinese version, partial). Mismatched probes are framed in rectangles. [file Image_3.tif]

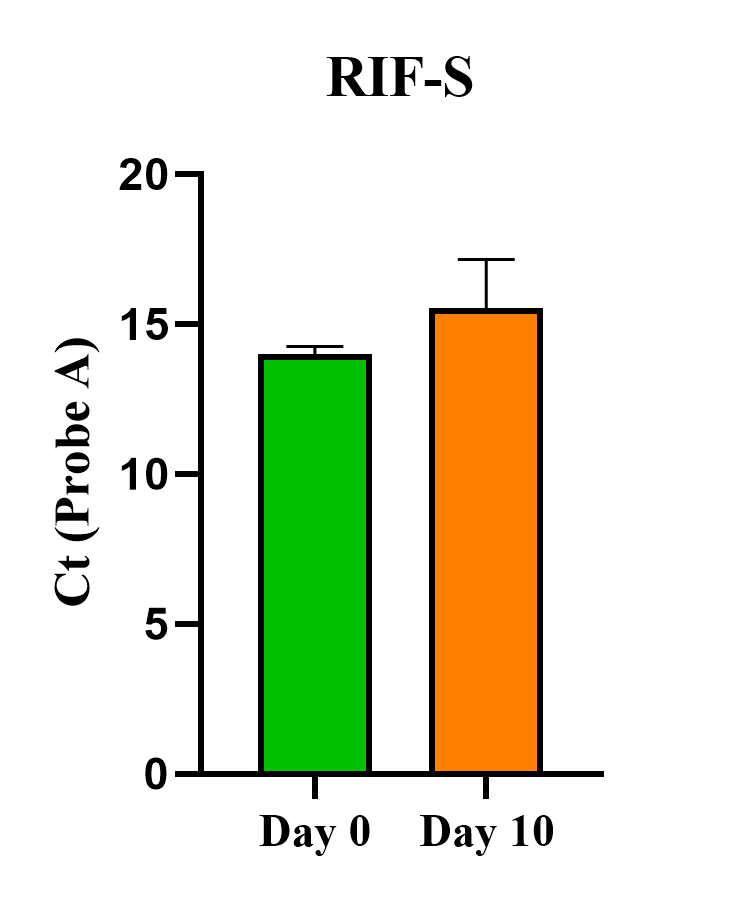

Supplement: Supplementary Figure 4 — Stability of specimens at 37°C. [file Image_4.tif]

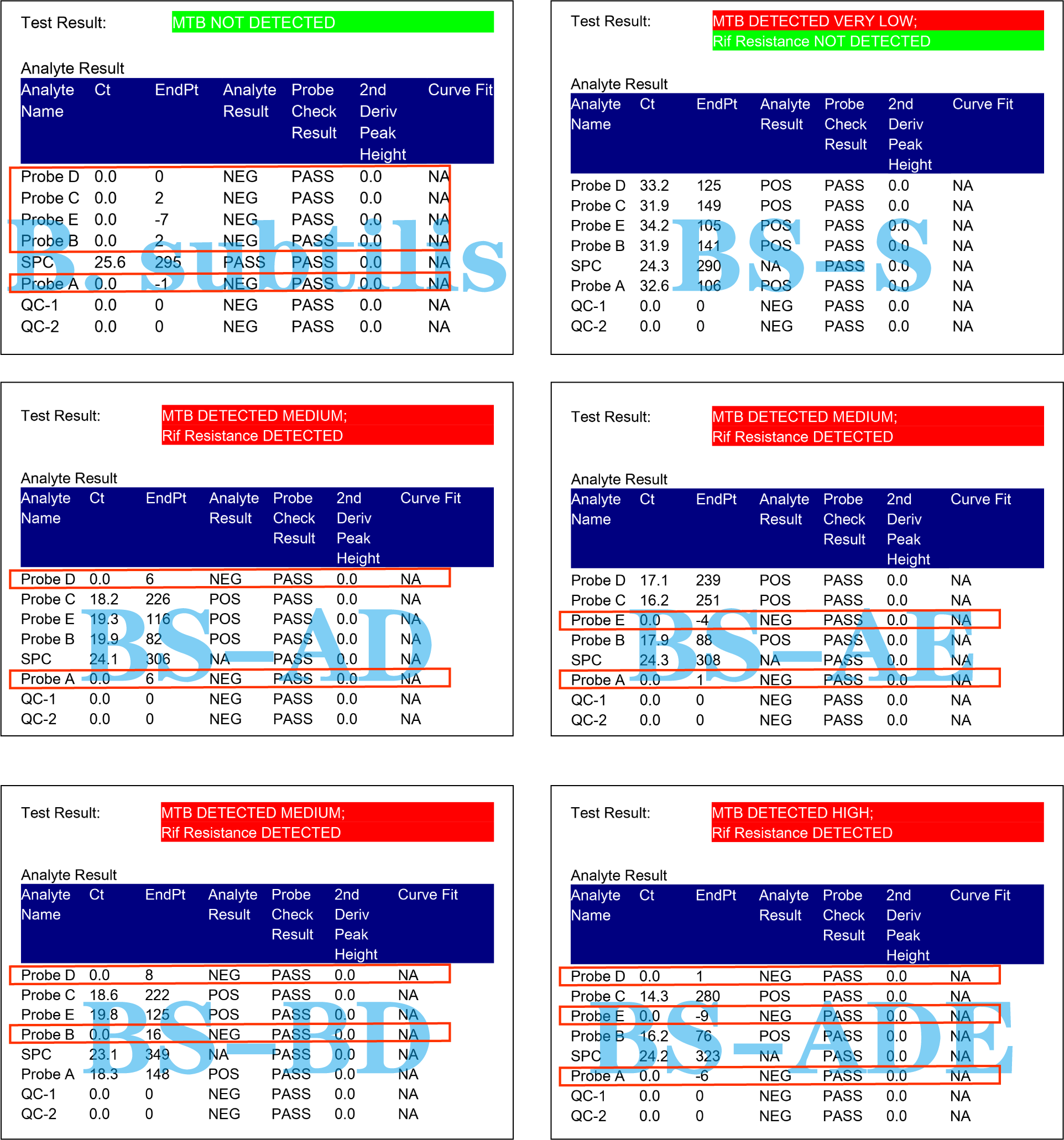

Supplement: Supplementary Figure 5 — Xpert MTB/RIF assay results of the library based on B. subtilis (GeneXpert Infinity System, partial). Mismatched probes are framed in rectangles. [file Image_5.tif]
