# Supplementary material for: Construction and application of a heterogeneous quality control library for the Xpert MTB/RIF assay in tuberculosis diagnosis
Source: Front Cell Infect Microbiol. 2023 Mar 17;13:1128337. doi: 10.3389/fcimb.2023.1128337 (PMC10063913; doi:10.3389/fcimb.2023.1128337)
Supplement: Supplementary file 6 [file Table_1.docx]

Supplementary Table S1 Synthesis fragments

| MTB-RIF-S |
| --- |
| gccgcctgcgtacggtcggcgagctgatccaaaaccagatccgggtcggcatgtcgcggatggagcgggtggtccgggagcggatgaccacccaggacgtggaggcgatcacaccgcagacgttgatcaacatccggccggtggtcgccgcgatcaaggagttcttcggcaccagccagctgagccaattcatggaccagaacaacccgctgtcggggttgacccacaagcgccgactgtcggcgctggggcccggcggtctgtcacgtgagcgtgccgggctggaggtccgcgacgtgcacccgtcgcactacggccggatgtgcccgatcgaaacccctgaggggcccaacatcggaattcaagcttcaagtcgaacggaaaggtctcttcggagatactcgagtggcgaacgggtgagtaacacgtgggtgatctgccctgcacttcgggataagcctgggaaactgggtctaataccggataggaccacgggatgcatgtcttgtggtggaaagcgctttagcggtgtgggatgagcccgcggcctatcagcttgttggtggggtgacggcctaccaaggcgacgacgggtagccggcctgagagggtgtccggccacactgggactgagatacggcccagactcctac |
| MTB-RIF-R |
| gccgcctgcgtacggtcggcgagctgatccaaaaccagatccgggtcggcatgtcgcggatggagcgggtggtccgggagcggatgaccacccaggacgtggaggcgatcacaccgcagacgttgatcaacatccggccggtggtcgccgcgatcaaggagttcttcggcaccagccagctgagccaattcatggtccagaacaacccgctgtcggggttgacctacaagcgccgactgttggcgctggggcccggcggtctgtcacgtgagcgtgccgggctggaggtccgcgacgtgcacccgtcgcactacggccggatgtgcccgatcgaaacccctgaggggcccaacatcggaattcaagcttcaagtcgaacggaaaggtctcttcggagatactcgagtggcgaacgggtgagtaacacgtgggtgatctgccctgcacttcgggataagcctgggaaactgggtctaataccggataggaccacgggatgcatgtcttgtggtggaaagcgctttagcggtgtgggatgagcccgcggcctatcagcttgttggtggggtgacggcctaccaaggcgacgacgggtagccggcctgagagggtgtccggccacactgggactgagatacggcccagactcctac |
