# Supplementary material for: Construction and application of a heterogeneous quality control library for the Xpert MTB/RIF assay in tuberculosis diagnosis
Source: Front Cell Infect Microbiol. 2023 Mar 17;13:1128337. doi: 10.3389/fcimb.2023.1128337 (PMC10063913; doi:10.3389/fcimb.2023.1128337)
Supplement: Supplementary file 7 [file Table_2.docx]

Supplementary Table S2 Primers used for polymerase chain reaction-based site-directed mutagenesis.

| Primers | Sequence (5′ to 3′) |
| --- | --- |
| MA-F | TTCTTCGGCACCAGCCAGCAGAGCCAATTCATGGACCAGAACAACCCGCTGTCGGGG |
| MA-R | CTGGTCCATGAATTGGCTCTGCTGGCTGGTGCCGAAGAACTCCTTGATCGCGGCGACC |
| MB-F | AGCTGAGCCAATTCATGGTCCAGAACAACCCGCTGTCGGGGTTGACCCACAAGCGCCG |
| MB-R | CGACAGCGGGTTGTTCTGGACCATGAATTGGCTCAGCTGGCTGGTGCCGAAGAACTCCT |
| MC-F | ATTCATGGACCAGAACAACACGCTGTCGGGGTTGACCCACAAGCGCCGACTGTCGGCGC |
| MC-R | TGGGTCAACCCCGACAGCGTGTTGTTCTGGTCCATGAATTGGCTCAGCTGGCTGGTGCC |
| MD-F | CAGCTGAGCCAATTCATGGACCAGAACAACCCGCTGTCGGGGTTGACCTACAAGCGCCG |
| MD-R | CGACAGCGGGTTGTTCTGGTCCATGAATTGGCTCAGCTGGCTGGTGCCGAAGAACTCCT |
| ME-F | ACCCACAAGCGCCGACTGTTGGCGCTGGGGCCCGGCGGTCTGTCACGTGAGCGTGCCGG |
| ME-R | ACCGCCGGGCCCCAGCGCCAACAGTCGGCGCTTGTGGGTCAACCCCGACAGCGGGTTG |
| MBD-F | CAGCTGAGCCAATTCATGGTCCAGAACAACCCGCTGTCGGGGTTGACCTACAAGCGCCG |
| Primer 1 | TTACTACCGCACTGCTGGCAGGATCTAGATATCGCCGCCTGC |
| Primer 2 | TAGGAGTCTGGGCCGTATCT |
