# Supplementary material for: Construction and application of a heterogeneous quality control library for the Xpert MTB/RIF assay in tuberculosis diagnosis
Source: Front Cell Infect Microbiol. 2023 Mar 17;13:1128337. doi: 10.3389/fcimb.2023.1128337 (PMC10063913; doi:10.3389/fcimb.2023.1128337)
Supplement: Supplementary file 8 [file Table_3.docx]

Supplementary Table S3 Mutant strains based on *E. coli.*

| Strains | Plasmids | Templates | Primers (see Table S2) |
| --- | --- | --- | --- |
| RIF-S | pRIF-S | \ | \ |
| RIF-BDE | pRIF-BDE | \ | \ |
| RIF-A | pRIF-A | pRIF-S | MA-F&MA-R |
| RIF-B | pRIF-B | pRIF-S | MB-F&MB-R |
| RIF-C | pRIF-C | pRIF-S | MC-F&MC-R |
| RIF-D | pRIF-D | pRIF-S | MD-F&MD-R |
| RIF-E | pRIF-E | pRIF-S | ME-F&ME-R |
| RIF-BE | pRIF-BE | pRIF-BDE | MB-F&MB-R |
| RIF-DE | pRIF-DE | pRIF-BDE | MD-F&MD-R |
